# 3D Bioprinting Mesenchymal Stem Cell-Derived Neural Tissues Using a Fibrin-Based Bioink

**DOI:** 10.3390/biom11081250

**Published:** 2021-08-21

**Authors:** Milena Restan Perez, Ruchi Sharma, Nadia Zeina Masri, Stephanie Michelle Willerth

**Affiliations:** 1 Department of Biomedical Engineering, University of Victoria, Victoria, BC V8W 2Y2, Canada; milenarestan@hotmail.com; 2 Department of Mechanical Engineering, University of Victoria, Victoria, BC V8W 2Y2, Canada; ruchis0983@gmail.com; 3 Division of Medical Sciences, University of Victoria, Victoria, BC V8W 2Y2, Canada; nadiam@uvic.ca

**Keywords:** 3D bioprinting, fibrin, small molecules, neural tissues, stem cells

## Abstract

Current treatments for neurodegenerative diseases aim to alleviate the symptoms experienced by patients; however, these treatments do not cure the disease nor prevent further degeneration. Improvements in current disease-modeling and drug-development practices could accelerate effective treatments for neurological diseases. To that end, 3D bioprinting has gained significant attention for engineering tissues in a rapid and reproducible fashion. Additionally, using patient-derived stem cells, which can be reprogrammed to neural-like cells, could generate personalized neural tissues. Here, adipose tissue-derived mesenchymal stem cells (MSCs) were bioprinted using a fibrin-based bioink and the microfluidic RX1 bioprinter. These tissues were cultured for 12 days in the presence of SB431542 (SB), LDN-193189 (LDN), purmorphamine (puro), fibroblast growth factor 8 (FGF8), fibroblast growth factor-basic (bFGF), and brain-derived neurotrophic factor (BDNF) to induce differentiation to dopaminergic neurons (DN). The constructs were analyzed for expression of neural markers, dopamine release, and electrophysiological activity. The cells expressed DN-specific and early neuronal markers (tyrosine hydroxylase (TH) and class III beta-tubulin (TUJ1), respectively) after 12 days of differentiation. Additionally, the tissues exhibited immature electrical signaling after treatment with potassium chloride (KCl). Overall, this work shows the potential of bioprinting engineered neural tissues from patient-derived MSCs, which could serve as an important tool for personalized disease models and drug-screening.

## 1. Introduction

Currently, animal models are used for disease modeling and in the drug-discovery process [1]. However, these models can be improved upon to accurately identify the efficacy and toxicity of potential drug targets. Additionally, they cannot capture the complexities and variability of human-neural tissues, which are necessary for accurate models [1,2,3,4]. One alternative to animal models is to use human-derived stem cells in combination with 3D bioprinting to generate neural tissues. For example, mesenchymal stem cells (MSCs) can be obtained from patients and reprogrammed to neural cells making them an effective source for personalized medicine. For this reason, this study focuses on 3D bioprinting MSCs that are reprogrammed into neuronal-like tissues to make a personalized neural model.

3D bioprinting has become a commonly used tool for neural tissue engineering applications as this process can rapidly and reproducibly generate tissue constructs based on specifications given in a digital file [5]. This process requires encapsulating the target cell type in a supportive bioink; however, due to the complexities and fragility of the brain and neural micro-environment, accurately reproducing the central nervous system (CNS) using this bioprinting process is more challenging [2,6]. Accordingly, the bioink formulation should mimic the extracellular matrix (ECM) to support cell-cell and cell-matrix interactions, cell proliferation, morphology, and differentiation [7]. Consequently, Abelseth et al. developed a novel fibrin-based bioink that supports the differentiation of human-induced pluripotent stem cells (hiPSCs) into neurons [8]. The novel bioink consisted of fibrinogen and alginate, which was then cross-linked with genipin, chitosan, thrombin, and calcium chloride [8]. This bioink supported the generation of hiPSC-derived neural tissues with high-cell viability and differentiation, as well as glioblastoma multiforme and astrocyte cells, which indicated its supportive properties for engineering a variety of neural tissues [8,9,10,11,12].

Several bioprinting techniques exist for generating 3D tissues including inkjet, extrusion, laser-assisted, and stereolithographic methods [13]. However, extrusion-based printers can print a variety of materials with varying viscosities—making it a conventional bioprinting strategy [2,13]. This digitally controlled process uses either a pressure or mechanical system to extrude biomaterials via a nozzle or syringe to form continuous fibers [2,13]. Nozzle-based bioprinters that require advanced microfluidic-chips can generate complex and highly biomimetic tissues by specifying the motion of the printhead and controlling pressure levels, which allows for microscale resolution and customizable cell and material patterning [13]. This printing process enabled the generation of tissues with the potential to mimic the complexities of human brain tissues. Neural cells are extremely responsive to environmental stresses, meaning that shear forces experienced by the cells during the bioprinting process must be minimized to avoid low cell viability and functionality [8,9,14]. Aspect Biosystems’ RX1 bioprinter (Aspect Biosystems, Vancouver, BC, Canada) uses a microfluidic printhead made of polydimethylsiloxane (PDMS), also known as a Lab-On-a-Printer (LOP^TM^), to deposit the bioink and crosslinker in a coaxial arrangement, where the crosslinker surrounds the bioink as it passes through the print nozzle [15,16]. This unique arrangement allows for the bioink to polymerize prior to being deposited, limiting the amount of shear stress experienced by the cells during extrusion, making it an effective technique for bioprinting stem cell-derived tissues [8,9,10,11,12,15,16].

Stem cells can both replicate to more stem cells and differentiate into a variety of cells, making them a powerful tool for 3D bioprinting [17]. MSCs are multipotent adult stem cells that can be derived from bone-marrow, umbilical cord, skin, and adipose tissue [18]. MSCs can directly differentiate into mesodermal lineages and transdifferentiate into non-mesodermal lineages such as neurons [18,19,20,21]. For example, Trzaska et al. [22] differentiated bone marrow MSCs into DNs, in 2D cell culture, for 12 days by using fibroblast growth factor 8 (FGF-8), basic fibroblast growth factor (bFGF), brain-derived neurotrophic factor (BDNF), and sonic hedgehog (SHH). Adipose-derived MSCs are the most promising for neural tissue engineering as they are easy to acquire in large numbers, and their robust nature allows them to survive the shear stresses during bioprinting [23,24]. During the neural induction process, adipose-derived MSCs also exhibit greater proliferation rates, increased neural marker expression and electrophysiological activity, in comparison to MSCs derived from other tissues [21]. Additionally, the ease of acquiring MSCs from a patient in comparison to the process required to generate hiPSCs, makes them a valuable tool for engineering personalized neural tissue models for both drug discovery and disease-modeling applications [25].

Multiple studies have 3D bioprinted scaffolds which are later seeded with MSCs for cartilage and bone tissue engineering [26,27,28,29]. However, MSC-laden bioinks have yet to be used for bioprinting neural tissues. Accordingly, here we 3D bioprinted MSCs using our fibrin-based bioink into half spheres to resemble the brain. Then, these MSCs were treated with factors to induce their differentiation into DNs based on the protocol by Trzaska et al. [22], with changing SHH to its agonist (purmorphamine), and the addition of LDN193189 (LDN) and SB431542 (SB). We then characterized these constructs after twelve days of culture in terms of viability, differentiation state, dopamine release and electrophysiological activity.

## 2. Materials and Methods

### 2.1. Cell Culture Expansion of MSCs

Human adipose-derived MSCs (Cat. No. 7510, ScienCell^TM^, Carlsbad, CA, USA) were cultured in a fibronectin coated T75 flasks in MSC Medium (MSCM, Cat. No. 7501, ScienCell^TM^, Carlsbad, CA, USA), according to manufacturer’s instructions. Upon reaching 90% confluency, the cells were cryopreserved in Dulbecco’s Modified Ealy Medium (DMEM, Cat. No. 11960044, Sigma, St. Louis, MO, USA) containing 10% dimethyl sulfoxide (DMSO, Cat. No. 472301, Sigma, St. Louis, MO, USA) using a slow rate-controlled cooling protocol (stored at −80 °C overnight and then in liquid nitrogen (−135 °C)).

### 2.2. Bioink Preparation

All components of the bioink, crosslinker, and buffer solution were prepared based on the protocol by Abelseth et al. [8]. Briefly, for the bioink, fibrinogen (Cat. No. 341578, EMD Millipore, Burlington, MA, USA) was prepared at a concentration of about 50 mg/mL in tris-buffered saline (TBS) solution and sterilized using a 0.2 μm syringe filter. Sodium alginate (Cat. No. 180947, Sigma-Aldrich, St. Louis, MO, USA) was prepared at 2% *w/v* by reconstituting in distilled water and sterilized using a 0.2 μm syringe filter. Genipin (Cat. No. G4796, Sigma-Aldrich, St. Louis, MO, USA) was reconstituted at a concentration of 25 mg/mL in dimethyl sulfoxide solution (DMSO). The final concentration in the bioink for fibrinogen, sodium alginate, and genipin was 20 mg/mL, 0.5% *w*/*v*, and 0.3 mg/mL, respectively. Additionally, the bioink protocol by [8] was slightly modified by adding 0.5% of phenol red, premixed in TBS, to enable better visualization of the construct during the printing process. The MSCs were then thawed and mixed with the bioink at a concentration of 2 × 10^6^ per 1 mL of bioink. Furthermore, the crosslinking solution consisted of 20 mg/mL of calcium chloride (CaCl_2_) (Cat. No. C1016, Sigma-Aldrich, St. Louis, MO, USA), thrombin at 1.7 UM/mL (Cat. No. T7009, Sigma-Aldrich, St. Louis, MO, USA), and chitosan at 0.075% *w/v* (Cat. No. C3646, Sigma-Aldrich, St. Louis, MO, USA). Prior to combining all components of the crosslinker, CaCl_2_ was prepared at a concentration of 20 mg/mL in sterile TBS. Thrombin was reconstituted at a concentration of 1000 UM/mL in sterile TBS. Chitosan was prepared at a concentration of 25 mg/mL using 1% acetic acid, and the pH was adjusted to 7.4 using β-glycerolphosphate (β-GP) (Cat. No. G9422, Sigma-Aldrich, St. Louis, MO, USA). The crosslinking solution was then prepared and sterilized by filtering using 0.2 μm syringe filters. The buffer solution consisted of a TBS solution.

### 2.3. Bioprinting MSCs

**Figure 1:**
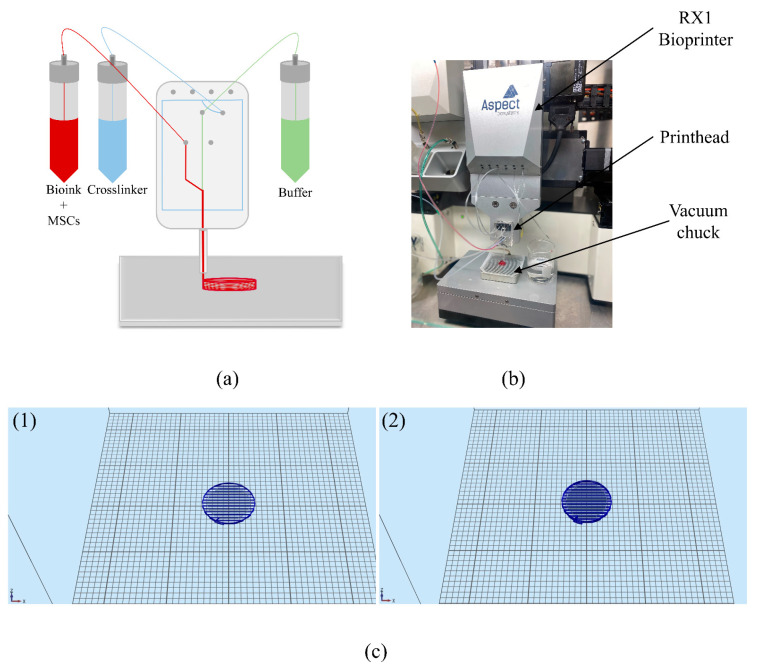
(**a**) Schematic representation of the Aspect Biosystems LOP^TM^ print-head technology; (**b**) Lay-out of bioprinter while printing constructs. (**c**) infill pattern of bioprinted constructed; (**1**) is one layer and (**2**) is two layers.

All the bioprinting was performed under sterile conditions. The bioink, buffer, and crosslinker were connected to the LOP^TM^ printhead (Aspect Biosystems, Vancouver, BC, Canada) via tubing as done previously [8]. Figure 1 depicts the set-up of the different components to the microfluidic printhead. The flow rate of the bioink and crosslinker were controlled by having specific pressures for both channels to allow enough time for the crosslinking reaction to occur in the nozzle, prior to being deposited on the vacuum chuck. Additionally, the pressures and printing speed were optimized and kept consistent throughout the printing process to produce homogenous constructs with even cell distribution, as shown in previous work [30]. The pressure of the crosslinker, bioink and buffer were 60 mbar, 50 mbar, and 100 mbar, respectively. The printing speed was 25 mm/s which allowed for four half-spherical constructs to be printed layer-by-layer in under five minutes. The bioprinted constructs were then carefully transferred using a sterile spatula to a 12-well cell culture plate coated with poly-L-ornithine (PLO, Cat. No. P4957, Sigma, St. Louis, MO, USA) and laminin (Cat. No. L2020, Sigma, St. Louis, MO, USA), containing MSC media (Cat. No. 7501, ScienCell^TM^, Carlsbad, CA, USA). Three replicates were printed for both control and experiment groups. The tissue design, which can be seen in Figure 1c, consists of 7 layers, and a 40% rectilinear infill pattern. This design was chosen according to previous experiments performed by our group, where the mechanical and physical properties were analyzed. In this study, the elastic moduli, storage and loss modulus, viscosity, and shear rates were measured. The results from these experiments confirmed that the bioprinted tissues possessed mechanical properties that were similar to native neural tissue. Furthermore, the elastic modulus was around 1 kPa, which is the optimal stiffness for neuronal differentiation [31]. Physical properties such as porosity, microstructure, swelling, and biodegradability were also analyzed. The tissues had a porosity of 66.1 ± 3.2%, indicating that there is adequate diffusion of nutrients and gases, and removal of metabolic waste, promoting differentiation and proliferation [31]. The degradation ratio was 27.8 ± 0.6%, which is sufficient for short-term experiments [31]. However, the study also found that incorporating microspheres into the bioink lowered the degradation ratio to 15.9 ± 1.0% [31]. Thus, if longer experiments are performed in the future, it would be ideal to incorporate drug-releasing microspheres. These results are promising since typically, fibrin polymerized by thrombin and CaCl_2_ degrades in one to two weeks due to proteases released from the incorporated cells [31]. However, the addition of genipin creates a multi-material scaffold by crosslinking with fibrin and chitosan, which stabilizes the bioprinted tissue.

### 2.4. Cell Culture Conditions

**Figure 2:**
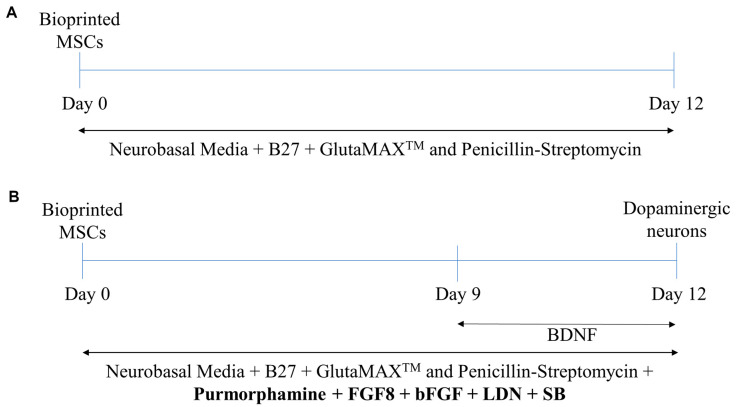
Schematic of cell culture period for (**A**) control group and (**B**) experimental group.

In addition to the 3D constructs, a 2D cell culture was prepared by coating a 12-well plate with PLO (Cat. No. P4957, Sigma, St. Louis, MO, USA) and laminin (Cat. No. L2020, Sigma, St. Louis, MO, USA), and 1 × 10^5^ MSCs per well were seeded. The 3D constructs and 2D cell cultures were incubated in MSC media (Cat. No. 7501, ScienCell^TM^, Carlsbad, CA, USA) overnight, at 37 °C and 5% CO_2_. On day 0 the following were treated: (a) control cells were treated with 2 mL of Neurobasal Media (NBM) (Cat. No. 21103049, ThermoFisher, Waltham, MA, USA), containing 2% B27 (Cat. No. 17504001, ThermoFisher, Waltham, MA, USA), 1% GlutaMAX^TM^ (Cat. No. 35050061, ThermoFisher, Waltham, MA, USA) and 1% Penicillin-Streptomycin (P/S, Cat. No. P4333, Sigma-Aldrich, St. Louis, MO, USA)—a protocol for basic MSC cell culture; and (b) the experimental cells were cultured in the same medium formulation with the addition of 250 ng/mL of purmorphamine (Cat. No. SML0868, Sigma-Aldrich, St. Louis, MO, USA), 100 ng/mL of FGF8 (Cat. No. 423-F8-025, R&D Systems, Minneapolis, MN, USA), 50 ng/mL of bFGF (Cat. No. 233-FB-010, R&D Systems, Minneapolis, MN, USA), 100 nM of LDN-193189 (Cat. No. 72147, STEMCELL Technologies, Vancouver, BC, Canada), and 10 µM of SB431542 (Cat. No. 72232, StemCell Technologies, Vancouver, BC, Canada) to induce the differentiation process. The cells were then incubated at 37 °C with 5% CO_2_ for 9 days, with no media changes in between. On day 9, 50 ng/mL of BDNF (Cat. No. 450-02, PeproTech, Rocky Hill, NJ, USA) was directly added to the experimental cells, without changing the media. They were then incubated for an additional three days, for a total of 12 days. A schematic of the cell culture period can be seen in Figure 2.

### 2.5. Cell Viability

Viability was assessed on the following days: (i) 0 (immediately after printing); (ii) day 9 (prior to the addition of BDNF); and (iii) day 12 using the LIVE/DEAD^TM^ Viability/Cytotoxicity kit (Cat. No. L3224, Thermo Fisher, Waltham, MA, USA). First, the medium was removed from the constructs followed by a wash with Dulbecco’s phosphate-buffered saline (DPBS). The DPBS was then removed, and the constructs were treated with 0.05% Calcein AM and 0.2% ethidium homodimer-1 in DPBS as per the manufacturer’s instructions. The constructs were then incubated at 37 °C with 5% CO_2_ for 30 min. Following this, the day 0 and day 9 constructs were imaged using a Leica DMI300 B microscope with an X-Cite Series 120Q fluorescent light source (Excelitas Technologies), a QImaging RETIGA 2000R camera at 10× magnification, and images were captured using the Qcapture Software 2.9.12. On day 12, the constructs were imaged using the FIPS-Zeiss Confocal Laser Scanning Microscope at 10 × magnification. Viability was quantified by imaging one spot on the construct and taking 15 images in varying Z-planes. A Z-projection was then created using ImageJ V1.52a, and the number of live and dead cells were counted using the ImageJ software to determine the viability throughout the construct.

### 2.6. Immunocytochemistry

Immunocytochemistry (ICC) was performed on both 3D and 2D cultures on day 12 to visualize the early neuronal marker TUJ1, and dopamine-specific marker tyrosine hydroxylase (TH). The media was removed, the cells were washed twice with PBS, and fixed with 10% formalin and incubated at room temperature for 10 min (for 2D culture) and 20 min (for 3D culture). Formalin was removed, cells were washed twice with PBS, with 2 min incubations at room temperature. The formalin was then removed, the cells were washed with PBS twice, and permeabilized with 0.1% Triton-X (Cat. No. HT501128, Sigma, St. Louis, MO, USA) and incubated at room temperature for 10 min (for 2D culture) and 45 min (for 3D culture). The Triton-X was removed, and the constructs were blocked with 5% normal goat serum (NGS) (Cat. No. ab7481, Abcam, Cambridge, UK) and incubated for 1–2 h, at room temperature. Then, two primary antibodies were added: anti-TH (Cat. No. ab112, Abcam, Cambridge, UK) at 1:500 and anti-TUJ1 (Cat. No. MAB1195, R&D Systems, Minneapolis, MN, USA) at 1:100 dilution in 5% NGS, incubated at 4 °C overnight with shaking at 100 rpm, and washed 3 times with PBS after incubation. Then, the secondary antibodies, goat anti-mouse (Alexa Fluor^®^ 488) (Cat. No. D1306, ThermoFisher, Waltham, MA, USA) and goat anti-rabbit (Alexa Fluor^®^ 568) (Cat. No. A11011, ThermoFisher, Waltham, MA, USA), were added at a 1:200 dilution in 5% NGS and incubated for 2 h at room temperature with shaking at 100 rpm. Cells were then washed twice with PBS. Lastly, the cells were stained with 300 nM of DAPI (Cat. No. D1306, ThermoFisher, Waltham, MA, USA) diluted in PBS, incubated for five minutes at room temperature, followed by two washes with PBS. The cells were imaged using the FIPS-Zeiss Confocal Laser Scanning Microscope. Following this, the number of DAPI-positive, TUJ1-positive, and TH-positive cells was counted using the ImageJ V1.52a software. Briefly, the cells were analyzed by first uploading the images to ImageJ, subtracting the background fluorescent as much as possible, adjusting the threshold until only the cells were visible, performing watershed segmentation, and analyzing the number of particles. Lastly, the number of TUJ1-positive and TH-positive cells were divided by the DAPI-positive cells to determine the percentage of cells that expressed each neuronal marker.

### 2.7. Dopamine Release

The cell culture supernatant was collected from 2D and 3D cultures, for both control and experimental group on day 12. These were then analyzed for the concentration of dopamine released using a dopamine-specific enzyme-linked immunoassay (ELISA) kit by following the manufacturer’s instructions (Cat. No. KA3838, Abnova, Taipei, Taiwan). The output absorbance was read using the TECAN infinite M200 Pro microplate reader set to 450 nm with a 635 nm reference wavelength. Afterward, a standard curve was obtained by plotting the mean absorbance readings (*n* = 3) on the *y*-axis (linear) against the corresponding standard concentrations (logarithmic) on the *x*-axis. A Spline curve fit was then obtained using Prism 5 (GraphPad) statistical software, and the unknown concentration values were interpolated for each unpaired absorbance reading (Table S1). This assay is a competitive assay so as absorbance values decrease, the concentration increases.

### 2.8. Electrical Properties

The electrophysiological activity of the DNs, for both the 2D and 3D cultures, was measured by using a voltage sensitive dye on the TECAN infinite M200 Pro microplate reader. This method was used since the 3D tissues could not be patch-clamped. This analysis was performed on three replicates for both control and experimental groups, and three cell-free constructs (3D) or blank wells (2D) which acted as a control for background fluorescence. First, a 1:1 ratio of FLIPR Membrane Potential Assay Kit Blue (Cat. No. R8042, Molecular Devices, San Hose, CA, USA) was added to the cells and incubated in the dark for 45 min at 37 °C with 5% CO_2_. The plate was then read by the microplate reader set to read fluorescent emission at 560 nm and take 25 reads from each well in 5 × 5 grid [32]. After the initial readings, KCl was added at a concentration of 56 mM, and the 3D constructs and 2D experiments were incubated for 30 min at 37 °C with 5% CO_2_. After incubation, the plates were read using the microplate reader under the same conditions as above. All data were analyzed using excel and equations set out in the protocol by Robinson et al. [32].

### 2.9. Statistical Analysis

For cell viability and electrophysiology experiments, a one-way ANOVA and Turkey post-hoc analysis was conducted, using a confidence level of 95% (*p* < 0.05). For ICC results, a two-tailed Student’s *t*-test, with a confidence level of 95% (*p* < 0.05), was conducted to determine the statistical significance between the control and experiment groups. All statistical analyses were conducted on the Prism 5 (GraphPad) statistical software.

## 3. Results

### 3.1. Characterization of the Bioprinted Constructs

**Figure 3:**
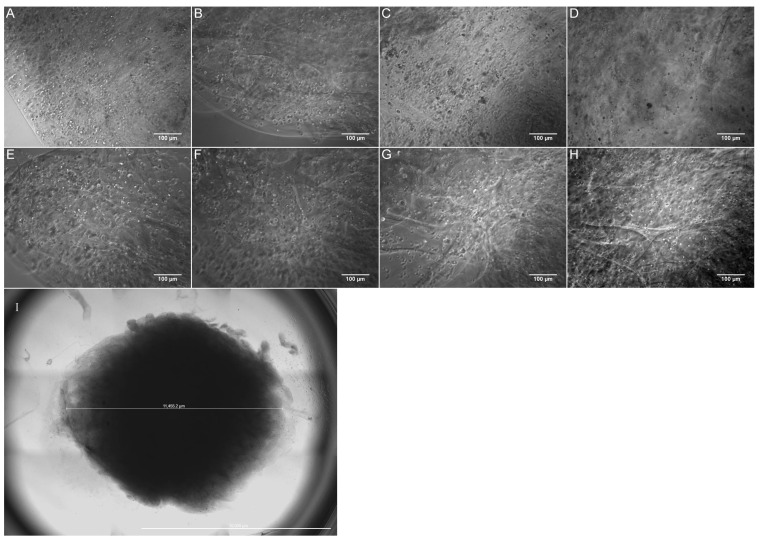
Phase contrast images of bioprinted half-spherical construct (**I**). Control group (**A**–**D**) and experimental group (**E**–**H**). Images were taken on day 0 (**A**,**E**), day 4 (**B**,**F**), day 9 (**C**,**G**) and day 12 (**D**,**H**). All images were taken at a single depth, representing one layer of the construct. Scale bar represents 100 µm (**A**–**H**) and 10 mm (**I**).

The 3D structures were half-spherical, 11.5 ± 6.9 mm in diameter (*n* = 3), and approximately 2.5 mm in height (Figure 3I). Phase contrast images of the 3D-printed MSCs (Figure 3A–H) were taken on the following days: 0 (day tissues were printed), day 4, day 9 and day 12, at 10 × magnification. Figure 3A–H shows that MSCs were evenly distributed throughout the construct using a layer-by-layer method and rectilinear pattern. It can also be seen that as the constructs approached day 12, the control group showed more cell death in comparison to the experimental group. These results match the 2D cell culture experiment (Figure S1).

### 3.2. Cell Viability

**Figure 4:**
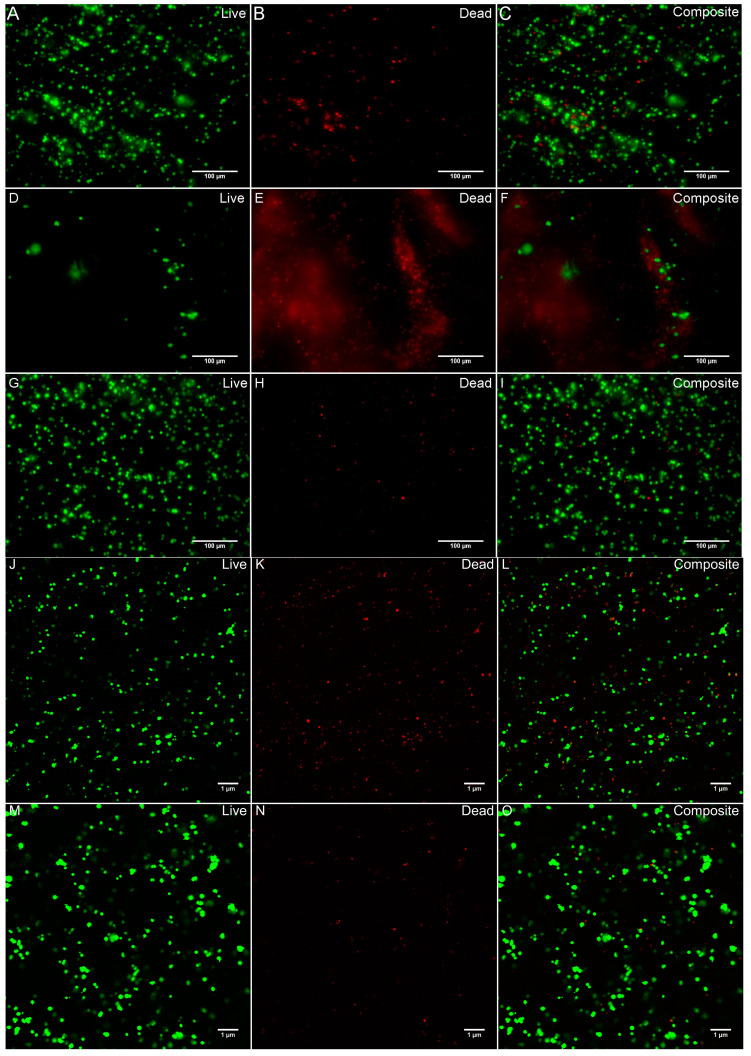

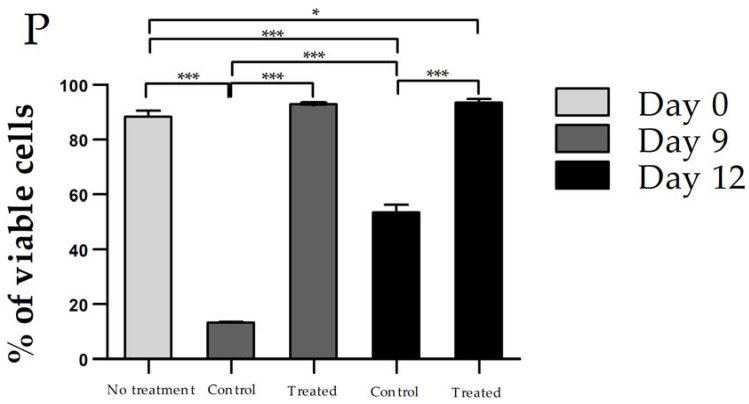
Cell viability images, using live/dead staining of bioprinted constructs on day 0 (**A**–**C**), day 9 for the control group (**D**–**F**) and experimental group (**G**–**I**), and day 12 for control (**J**–**L**) and experimental group (**M**–**O**). Images were quantified and values are given as average ± standard deviation (*n* = 3). One-way ANOVA and Turkey post-hoc analysis was conducted, using a confidence level of 95%; * *p* < 0.05; *** *p* < 0.001. Scale bar represents 100 µm for (**A**–**I**) and 1 µm for (**J**–**O**). (**P**) shows quantification of the viable cells present in the constructs at Day 0, 9, and 12.

Figure 4 shows representative images and quantified values of the constructs stained for viability on day 0, day 9, and day 12. Viability on day 0 was performed immediately after printing the constructs, on day 9 prior to adding BDNF, and on day 12 when the induction was completed. Cell viability on day 0 was 88.4 ± 1.3%, and on day 9 it was 13.4 ± 0.1% for the control group and 92.9 ± 0.2% for the experiment group. Finally, on day 12, cell viability was 53.4 ± 1.6% for the control group and 93.5 ± 0.7% for the experimental group. The decrease in viability for the control group in comparison to the experimental group on day 12 agrees with our 2D cultures, where the number of DAPI-positive cells was significantly less in the control group when compared to the experimental group (Figure S2F,J). While the same seeding density was used for both 2D groups, the control group contained (2.17 ± 0.04) × 10^5^ cells, whereas the experimental group contained (7.62 ± 0.76) × 10^5^ cells by day 12. These values were found to be significantly different after performing a two-tailed, unpaired, student *t*-test with a confidence level of 95%. To continue, the control group’s viability significantly increased on day 12 in comparison to day 9. However, the experimental group did not show any significant differences in cell viability between day 9 and 12. Lastly, a significant difference was found between the no-treatment group (day 0) and the control group on days 9 and 12, as well as with the experimental group on day 12.

### 3.3. Immunocytochemistry

**Figure 5:**
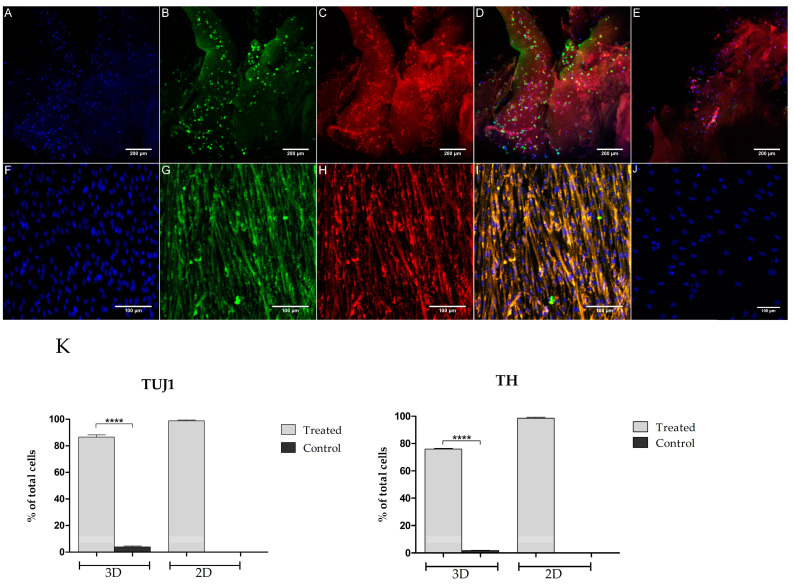
Immunocytochemistry of 3D (**A**–**E**) and 2D (**F**–**J**) cell cultures after 12 days of differentiation. In 3D, the experimental group (**A**–**D**) and control group (**E**) were imaged at 20×; In 2D, the experimental group (**F**–**I**) was imaged at 10 × and control group (**J**) was imaged at 20×. Scale bar represents 200 µm for 3D culture, and 100 µm for 2D culture. The quantitative results for the number of TUJ1-positive and TH-positive cells are seen in (**K**). A two-tailed Student’s *t*-test, with a confidence level of 95% (*p* < 0.05), was conducted to determine the statistical significance between the control and experiment groups; **** *p* < 0.0001.

Figure 5 shows the immunocytochemistry results from day 12 for the 2D and 3D cultures, for both control and experimental groups. When comparing the number of cells expressing neural cell marker to the number of nucleated cells (DAPI expression), the experimental group showed significantly more TH and TUJ1 expression than the control group (*p* < 0.0001). For the experimental and control groups, 86.67 ± 1.6% and 4.1 ± 0.6% of cells expressed TUJ1, respectively. TH expression in the experimental and control group was 75.9 ± 0.5% and 1.8 ± 0.2%, respectively. It was also found that there were no neurite extensions in the 3D culture, whereas in the 2D culture there was some neurite extension. To extend, for the 2D cultures, 87 ± 1.5% of the TUJ1-positive cells showed neurite extensions. Additionally, the 2D cultures showed more TUJ1-positive (98.8 ± 0.6%) and TH-positive cells (98.5 ± 0.2%) in comparison to the 3D cultures.

### 3.4. Dopamine Release

**Figure 6:**
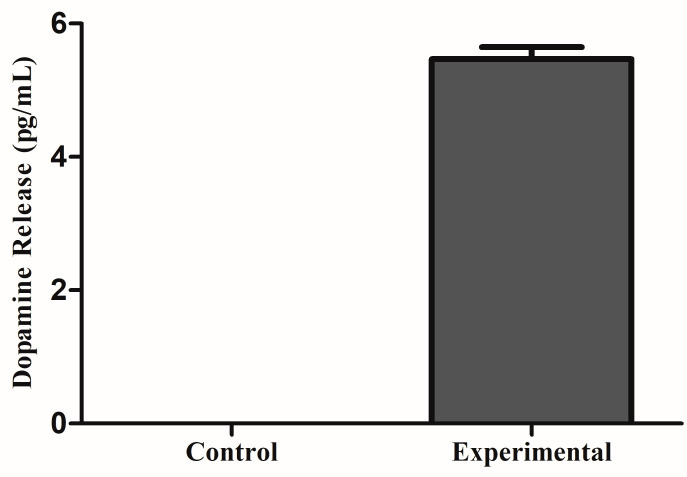
3D bioprinted neural tissues secrete dopamine in comparison to untreated 3D bioprinted tissues.

The dopamine released from the 3D experimental group, on day 12, was 5.47 ± 0.18 pg/mL (*n* = 3). This was significant considering that for all 2D samples and 3D controls, the absorbance readings were all above the standard curve, indicating that there was no dopamine release. These results can be found in Figure 6.

### 3.5. Electrophysiological Activity

**Figure 7:**
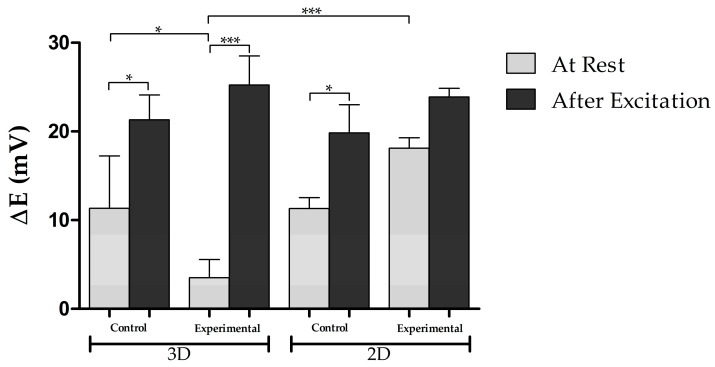
Electrical activity of 2D and 3D cultures (control and experimental) at resting membrane potential and after excitation with KCl. *n* = 3 for all groups. Values are given as average ± standard deviation. One-way ANOVA and Turkey post-hoc analysis was conducted, using a confidence level of 95%; * *p* < 0.05; *** *p* < 0.001.

The results of the electrophysiological properties of the bioprinted tissues and 2D cultures are shown in Figure 7. At rest, the experimental group had a potential energy of 3.5 ± 1.2 mV in 3D and 18.1 ± 0.6 mV in 2D, while the control group had a potential energy of 11.3 ± 3.4 mV in 3D and 11.3 ± 1.2 mV in 2D. For the 3D cultures, these results show that the experimental group had a hyperpolarized resting membrane potential (3.5 ± 1.2 mV) when compared to the control group (11.3 ± 3.4 mV), indicating that the experimental group was more electro-physically mature. In contrast, for the 2D cultures, the membrane potential was higher for the experimental group. This potentially indicates that the 3D environment allowed the cells to become more electro-physically mature. After excitation with KCl, the experimental group’s potential energy increased significantly to 25.3 ± 1.9 mV for the 3D cultures, and to 23.8 ± 1.0 mV for the 2D cultures. Furthermore, the control group’s action potential increased to 21.3 ± 1.6 mV for the 3D cultures, and 19.8 ± 3.2 mV for the 2D cultures. For the 3D cultures, there was a higher significant difference between the resting membrane potential and excited potential for the experimental group, since *p* < 0.05 for the control group and *p* < 0.001 for the experimental group. On the other hand, for the 2D cultures, no significant difference was found for the experimental group, but there was a significant difference in the control group (*p* < 0.05). Lastly, when comparing the membrane potentials between the 3D and 2D experiments, at rest and excitation, there was only a significant difference found between the experimental groups resting membrane potential (*p* < 0.001).

## 4. Discussion

In this study, MSC-derived neural-like tissues were successfully bioprinted using our fibrin-based bioink and the RX1 bioprinter. To begin, our 2D studies showed that the group treated with small molecules LDN193189 and SB431542 had more TH-positive cells, indicating that neuron maturation improved in the presence of these small molecules. For this reason, the printed constructs were differentiated for 12 days using growth factors with the addition of LDN and SB. Immediately after printing the constructs, on day 0, the percentage of viable cells was 88.4 ± 1.3%. After 9 days in culture, the percentage of viable cells was 13.4 ± 0.1% for the control group and 92.9 ± 0.2% for the experimental group. Lastly, on day 12, the percentage of viable cells was 53.4 ± 1.6% for the control group and 93.5 ± 0.7% for the experimental group. Comparisons between the control and experimental groups on days 9 and 12, showed a significant decrease in the percentage of viable cells for the control group. These results agree with our 2D experiments, where the number of DAPI-positive cells in the control group significantly diminished in comparison to the experimental group on day 12. This could be attributed to the lack of media changes throughout the 12 days of differentiation, which was done because [22] noted that there was the production of factors that contribute to the generation of functional DNs. Additionally, the viability results also imply that the growth factors and small molecules may aid in cell viability. However, when comparing day 0 to day 12 of the experimental group, the percentage of viable cells increased significantly by 5.2 ± 1.5%. The lower percentage on day 0 in comparison to days 9 and 12 could be attributed to the inevitable environmental stresses experienced by the cells during the bioprinting process. In addition, after 12 days of differentiation, with no disturbances, the cells had a chance to proliferate, and the number of viable cells increased accordingly—these results also agree with our 2D cultures. Viability percentages from this experiment are similar to previous viability claims for 3D-printed NPCs, indicating that our fibrin-based bioink and 3D bioprinting technique not only supports NPCs but also MSCs [26,31].

ICC results of the 3D constructs showed that TH and TUJ1 cell expression per number of DAPI-positive cells was higher for the experimental group than the control group. However, no neurite extension was seen in the constructs in comparison to the neurite extensions seen in 2D cultures. This could be attributed to the auto-fluorescent properties of the bioink, which make it extremely challenging to image the cells and poor diffusion of the molecules and growth factors into the construct. In the future, it would be recommended to slice the construct and place it on coverslips prior to imaging. When studying the effects of LDN and SB in the 2D cultures, ICC results showed that TUJ1-positive cells were also TH-positive for the group that was treated with LDN and SB. Additionally, no significant difference was found between the TUJ1-positive and TH-positive cells. These results are an improvement from the Trzaska et al. [33] paper, where out of a >90% population of TUJ1-positive cells, 69% expressed TH. However, in the 3D experiments, out of 86.67 ± 1.6% of cells that expressed TUJ1, 75.9 ± 0.5% of cells expressed TH. This could be attributed to poor diffusion of the small molecules and growth factors into the construct. To extend, mechanical and physical property experiments by Sharma et al. [31] showed that the bioprinted tissues had a porosity of 66.1 ± 3.2%, which suggests that all the components of the media cannot diffuse through the construct and reach the cells. In contrast, in 2D experiments, the small molecules and growth factors settle at the bottom of the plate where the cells are attached and thus, no diffusion was necessary. A plausible solution to improve diffusion in 3D cultures would be to incorporate drug-releasing microspheres into the bioink.

Electrophysiology results showed that the resting membrane potential of the experimental and control groups, for the 3D cultures, were 3.5 ± 1.2 mV and 11.3 ± 3.4 mV, respectively. Additionally, for the 2D cultures, the resting membrane potential of the experimental and control groups were 18.1 ± 0.6 mV and 11.3 ± 1.2 mV, respectively. However, for the 2D cultures, there was no significant difference found between groups at the resting membrane potential. For the 3D cultures, since the potential of the control group was much higher than the experimental group, these results suggest that the experimental group was more electro-physically mature than the control group. Additionally, since the resting potential was significantly hyperpolarized in the 3D cultures but not in the 2D cultures, this suggests that the 3D environment allowed the cells to become more electro-physically mature. However, since the resting membrane potential of the cells was not close to the −70 mV found in vivo, these results indicate that immature electrical activity was occurring. After the cells were excited, the potentials of the experimental and control groups for the 3D cultures increased to 25.3 ± 1.9 mV and 21.3 ± 1.6 mV, respectively. For the 2D cultures, the membrane potential increased to 23.8 ± 1.0 mV for the experimental group, and 19.8 ± 3.2 mV for the control group. For the 3D culture readings, the experimental group had a higher significant difference between the excited membrane potential and resting membrane potential, in comparison to the control group, indicating that electrical stimulation in the experimental neural cultures was more significant. In contrast, for the 2D cultures, there was no significant difference found in the experimental group, which further indicates that the 3D environment allowed for greater neural maturation. It is important to note that stem cell-derived DNs require extended periods of culture before developing fully mature electrical activity in vitro. For example, Gonzales et al. [34] used guggulsterone (a natural steroid) to differentiate DNs from hiPSCs and found that electrical activity was not observed until 90 days post-differentiation. For immature electrical activity to occur in this short 12-day differentiation protocol, it suggests that the 3D microenvironment they were cultured in allowed for faster electrophysical maturation.

DNs secrete dopamine to regulate voluntary movement, for this reason their ability to release dopamine is an important physiological feature. In this study, the experimental group secreted 5.47 ± 0.18 pg/mL of dopamine after 12 days of differentiation. This was significant considering that for all 2D cultures and the 3D control group, there was no detectable dopamine secretion. The amount of dopamine secreted in this experiment was less than the amount reported by Gonzales et al. [34], where the amount of dopamine was about 1 ng/mL. However, these hiPSC-derived DNs secreted dopamine after 24 days of differentiation. Similarly, experiments by Robinson et al. [35] found that hiPSC-derived DNs secreted dopamine after 41 days of neural induction. These results suggest that the 3D microenvironment of the neural tissues allowed for faster DN development when compared to 2D cultures.

In future experiments, it would be beneficial to bioprint the cells with drug-releasing microspheres, thus allowing cells to receive a direct and controlled release of the drug rather than having the molecules diffuse through the construct to reach the cells. A study using microspheres was performed by De la Vega et al. [36], where purmorphamine (puro) and retinoic acid (RA) loaded microsphere were incorporated into cell aggregates to promote differentiation of iPSCs into mature motor neurons. After 35 days, their results showed extensive and organized neurite growth extension. Similarly, a study by Sharma et al. [12] showed that hiPSC-derived neural constructs containing guggulsterone microspheres improved neuronal differentiation and cell proliferation in comparison to constructs that were treated with soluble guggulsterone. A similar protocol could be followed for this experiment by incorporating microspheres into the MSC-laden bioink and following the printing methods previously described [8]. Lastly, it would be beneficial to perform a polymerase chain reaction as a future experiment to further verify marker expression and confirm cell differentiation.

With further maturation, these MSC-derived neural tissues could be useful as a disease modeling and drug-screening tool for Parkinson’s disease (PD). Patients who suffer from PD experience tremors, muscle rigidity, and imbalance due to the depletion of DNs in the substantia nigra which is necessary for voluntary movements [35,37,38]. Some treatments can alleviate these symptoms such as the drug levodopa but over time, this drug causes uncontrolled movement and cannot cease the degradation of DNs nor cure the disease [33,35,37,38]. We suggest that our neural tissues containing MSCs and fibrinogen derived from patients with PD could potentially serve as a more accurate personalized model to study the progression of PD. Additionally, with further maturation, these tissues could also be personalized for potentil implantation urposes. With this knowledge, we can better predict the efficacy, safety, and toxicity of potential drug targets for treating PD.

## 5. Conclusions

In this study, we successfully bioprinted MSC-derived neural tissues using a fibrin-based bioink and the RX1 bioprinter. Characterization of our neural tissues showed high cell viability, expression of early neuronal marker (TUJ1) and DN marker (TH), and dopamine release. Additionally, the tissues expressed immature electrical signaling when exposed to KCl. These neural tissues can be personalized by using PD patient-derived MSCs, making them a great tool for personalized disease-models and drug-screening.

## Data Availability

The data presented in this study is contained in this manuscript or is available on request from the corresponding author. The data that are not publicly available is because as we have shown representative images from all experiments in this paper and its supplementary figures.

## Supplementary Materials

The following are available online at https://www.mdpi.com/article/10.3390/biom11081250/s1, Figure S1: Phase contrast images of 2D cell culture experiment. (A–D) Group treated without LDN nor SB; (E–H) Group treated with LDN and SB. (I–L) Control group. Images were taken on day 1 (A,E,I) prior to treatment, day 4 (B,F,J), day 9 (C,G,K), and day 12 (D,H,L)., Figure S2: Immunocytochemistry of 2D cell culture. Group treated without LDN or SB (A–D), group treated with LDN and SB (F–I), and control group (E,J). All scale bars represent 100 µm, Table S1: Mean absorbance values (*n* = 3) and concentrations for all standards, from which the standard curve graph was derived from. For all samples (bolded), the concentrations were interpolated from the standard curve graph.
