# Supplementary material for: 3D Bioprinting Mesenchymal Stem Cell-Derived Neural Tissues Using a Fibrin-Based Bioink
Source: Biomolecules. 2021 Aug 21;11(8):1250. doi: 10.3390/biom11081250 (PMC8394541; doi:10.3390/biom11081250)

Supplemental Data

To determine the effects of LDN and SB in the differentiation of MSCs to DN, a 2D cell culture experiment was conducted where one group was treated with neurobasal media containing FGF8, FGFb, purmorphamine, B27, penicillin-streptomycin, GlutaMAX, and BDNF on day 9. Another group contained all the growth factors mentioned above, with the addition of 100 nM of LDN193189 and 10 μM of SB431542. The control group contained neurobasal medium, B27, GlutaMAX, and penicillin/streptomycin. All groups were cultured for 12 days with no media changes.

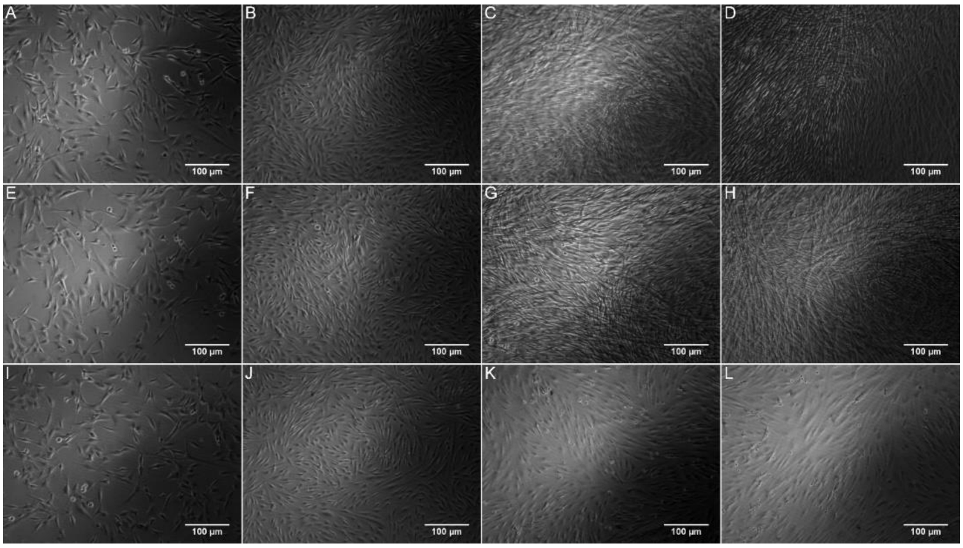

Figure S1. Phase contrast images of 2D cell culture experiment. (**A–D**) Group treated without LDN nor SB; (**E–H**) Group treated with LDN and SB. (**I–L**) Control group. Images were taken on day 1 (**A,E,I**) prior to treatment, day 4 (**B,F,J**), day 9 (**C,G,K**), and day 12 (**D, H, and L**).

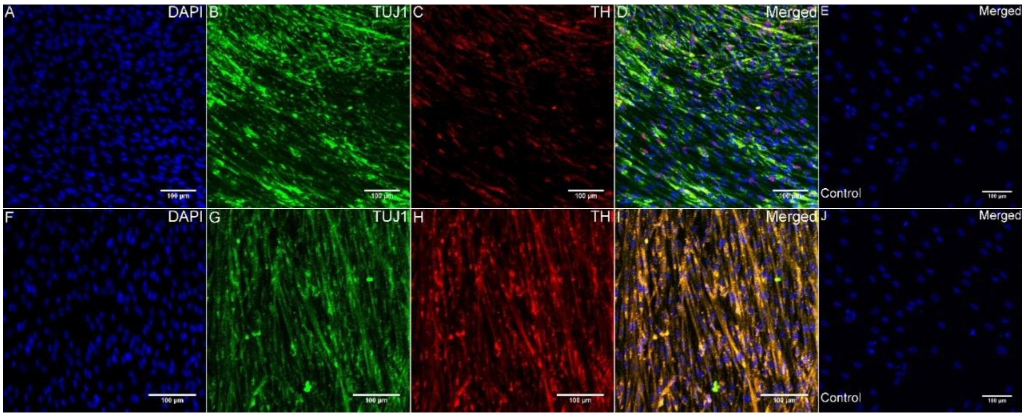

Figure S2. Immunocytochemistry of 2D cell culture. Group treated without LDN or SB (**A–D**), group treated with LDN and SB (**F–I**), and control group (**E,J**). All scale bars represent 100 μm.

Table S1. Mean absorbance values (n = 3) and concentrations for all standards, from which the standard curve graph was derived from. For all samples (bolded), the concentrations were interpolated from the standard curve graph.

Standards and Samples	Mean Absorbance [nm]	Concentration [ng/mL]
**Standard A**	1.804	0
**Standard B**	1.762	0.5
**Standard C**	1.634	1.5

**Standard D**	1.373	5
**Standard E**	0.999	20
**Standard F**	0.609	80
**2D Treated**	**1.836**	
**2D Control**	**1.806**	
**3D Treated**	**1.783**	**0.00547**
**3D Control**	**1.84**	

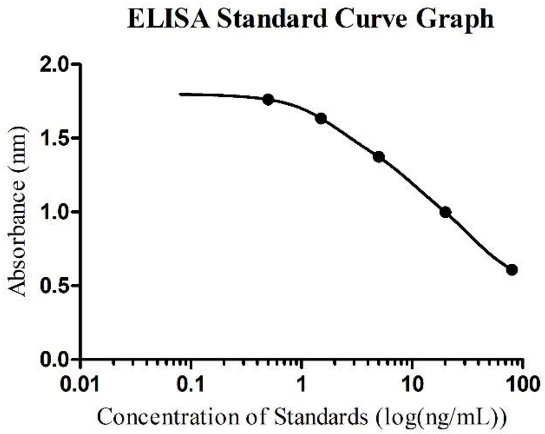

Supplement: Supplementary file 1 [file biomolecules-11-01250-s001.zip › biomolecules-1304099-supplementary.pdf]
